# Supplementary material for: Purified Gymnemic Acids from Gymnema inodorum Tea Inhibit 3T3-L1 Cell Differentiation into Adipocytes
Source: Nutrients. 2020 Sep 17;12(9):2851. doi: 10.3390/nu12092851 (PMC7551785; doi:10.3390/nu12092851)
Supplement: Supplementary file 1 [file nutrients-12-02851-s001.pdf]

Supplementary Information

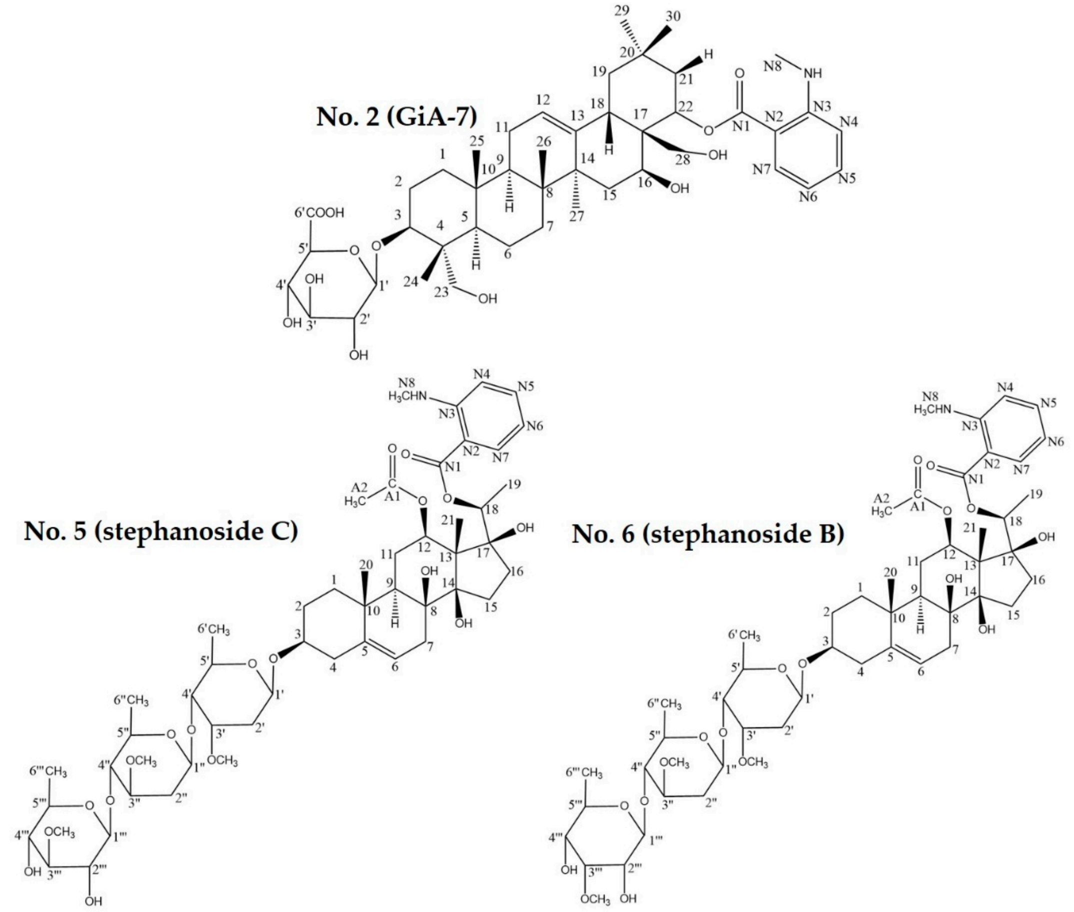

Figure S1. Purified gymnemic acids from *Gymnema inodorum* tea inhibit 3T3-L1 cell differentiation into adipocytes. The structural formulae of compounds 2, 5 and 6.
